# Serious Treatment Related Adverse Drug Reactions amongst Anti-Retroviral Naïve MDR-TB Patients

**DOI:** 10.1371/journal.pone.0058817

**Published:** 2013-04-03

**Authors:** Martha Van der Walt, Johanna Lancaster, Ronel Odendaal, Jeanne Garcia Davis, Karen Shean, Jason Farley

**Affiliations:** 1 Tuberculosis Epidemiology and Intervention Research Unit, South African Medical Research Council, Pretoria, South Africa; 2 Johns Hopkins University School of Nursing, Baltimore, Maryland, United States of America; McGill University, Canada

## Abstract

**Background:**

Globally treatment outcomes for multidrug-resistant *Mycobacterium tuberculosis* (MDR-TB) remain poor and this is compounded by high drug toxicity. Little is known about the influence of adverse drug reactions (ADRs) on treatment outcomes in South Africa.

**Methods:**

We evaluated the impact of severe ADRs among a prospective cohort of MDR-TB patients in South Africa (2000–2004). The HIV-infected study participants were anti-retroviral naïve.

**Results:**

Of 2,079 patients enrolled, 1,390 (66.8%) were included in this analysis based on known HIV test results (39.1% HIV-infected). At least one severe ADR was reported in 83 (6.9%) patients with ototoxicity being the most frequent ADR experienced (38.9%).

**Conclusions:**

We found that being HIV-infected but antiretroviral naïve did not increase occurrence of SADRs in patients on second-line anti-tuberculosis drugs. Early screening and proactive management of ADRs in this patient population is essential, especially given the rollout of decentralized care and the potential for overlapping toxicity of concomitant MDR-TB and HIV treatment.

## Introduction

One of the challenges facing patients treated with second-line drugs (SLD) is the toxic nature of these drugs [1]. This is exacerbated in settings with high co-infection with the human immunodeficiency virus (HIV) due to the potential overlap and additive drug toxicity of anti-retroviral treatment (ART) [2]–[4]. Adverse drug reactions (ADRs) are described in many MDR-TB cohort studies with varying profiles between different settings [5]–[8]. These studies provide limited insight into the severity of ADRs experienced and include relatively small numbers of patients with HIV co-infection. The South African tuberculosis epidemic, including that of drug-resistant TB disease, is typified by high HIV co-infection rates [9] and poor treatment outcomes of drug-resistant TB [10], [11].

An ADR is defined as: any unintended adverse response to a drug occurring at a therapeutic dose and resulting in either death, drug withdrawal, change in the administration of the frequency or dose of the drug, or, that no action is required. The severity of these reactions can range from mild, no intervention required, to the more severe or life-threatening (SADR) where drugs need to be withdrawn, either completely from the regimen or with re-introduction once the SADR has subsided. Proactive and vigilant management of SADRs among patients treated with SLDs are especially important as the access of MDR-TB cases to anti-retroviral treatment (ART) continues to expand and guidelines now recommending treatment of MDR-TB patients with ART regardless of their CD4 count [12].

We conducted a prospective DOTS-plus study to examine treatment outcomes among a large cohort across South Africa [13]. The purpose of the secondary analysis described here is to examine the serious adverse drug reaction profile due to second-line anti-tuberculosis treatment. HIV infection rates were high, but none of the patients received ART. We compared patients who experienced SADRs to those who did not, described the range of SADRs and assessed their impact on treatment outcomes.

## Methods

### Ethics Statement

This study was approved by the South African Medical Research Council's Ethics Committee, the nine Provincial Research Committees (Western Cape, Northern Cape, Eastern Cape, Gauteng, Mpumalanga, Kwa-Zulu Natal, Limpopo, North West, and Free State) and the Johns Hopkins University School of Public Health Institutional Review Board. Written informed consent was obtained from each study participant before enrolment and the informed consent process was approved by the respective ethical/review boards. Members of the clinical teams at the treatment centres, or the site-specific research coordinator were trained and capacitated to invite patients to participate in the study, obtain informed consent, complete the documentation as well as to countersign and provide MRC with the completed case record forms (CRFs). Data capturing from the case record forms was not done on those patients for whom a signed consent form was not available.

### Patient population, treatment and data collection

Between 2000 and 2004 adults, 18 years and above who presented with a culture proven bacteriological diagnosis of MDR-TB were consecutively enrolled in the DOTS-Plus study [13]. Enrollment took place at eleven MDR-TB treatment centres in all nine South African provinces. Only new MDR-TB patients were enrolled, and those who transferred out after cohort entry were later excluded.

Since 2000, most of the MDR-TB treatment centres in South Africa have used standardised programmatic management of MDR-TB (DOTS-Plus) [13]. A SLD regimen was used, consisting of a 4 month hospital-based intensive phase of pyrazinamide, ethambutol or cycloserine/terizidone (if resistant to ethambutol), ethionamide, ciprofloxacin/ofloxacin, and amikacin/kanamycin. This was followed by an additional 12 to 18 months, outpatient based continuation phase, in which the injectable agent and pyrazinamide were omitted. CRFs were prospectively completed by a clinician or nurse. The patient data collected included demographic data, baseline treatment and clinical information, as well as monthly treatment and clinical data. HIV testing was offered to all patients upon start of MDR-TB treatment, but ART has only been available to MDR-TB patients since late 2004, thus making this cohort ART naïve.

The CRF included an ADR screening tool which as well as listing the 16 most commonly identified drug reactions related to SLD treatment (Table 1), asked for information on severity, drug/s implicated and time of occurrence (week/month). The clinicians were required to record only serious ADRs, which were passively screened, either by patient self-reporting or through observation by the clinician/nurse. Screening was required weekly for in-patients, during the intensive phase where patients were under constant and close observation. Admission was advocated in settings where access to resources such as audiology screening was limited or for clinicians with limited previous experience of the use of second-line regimens. The tool was also designed according to the anticipated frequency of follow up visits under DOTS-Plus, i.e. weekly during intensive phase and monthly once the patient was discharged (follow up was continued for at least 16 months). Vigilant recording of SADRs was ensured by ongoing training of clinicians and regular auditing of CRFs to ensure that any regimen changes related to SADRs were appropriately recorded.

**Table 1:** Frequency of Serious Adverse Drug Reactions by HIV status and months of treatment.

Serious Adverse Drug Reaction	Total reactions N = 108 n(%)	HIV status of patients experiencing SADRs		Frequency of occurrence during months 1–4 n(%)	Drug implicated
		HIV- infected N = 50 n(%)	HIV- uninfected N = 58 n(%)		
Decreased Hearing	42(38.9)	20(40)	22(38)	41(38)	Kanamycin/Amikacin
Psychosis	16(14.8)	7(14)	9(15.5)	8(9.1)	Cycloserine/Terizidone/Ofloxacin
Dizziness	9(8.3)	3(6)	6(10)	8(9.1)	Kanamycin
Tinnitus	7(6.55)	3(6)	4(6.9)	6(6.8)	Kanamycin
Nausea	6(5.5)	2(4)	4(6.9)	2	Ethionamide
Vomiting	6(5.5)	4(8)	2(3.4)	4(4.5)	Ethionamide
Joint Pain	4(3.7)	1	3(5.2)	4(4.5)	-
Depression	4(3.7)	3(6)	1	1	Cycloserine/Terizidone
Rash	2(2.8)	2(4)	0	2	Kanamycin
Painful/Inflamed injection site	4(3.7)	1	3(5.2)	4(4.5)	Kanamycin
Epileptic fit	3(2.8)	2(4)	1	3(3.4)	Cycloserine/Terizidone/Ofloxacin
Tremors	1	1	0	1	Ofloxacin
Abscess	1	0	1	1	Kanamycin
Renal failure	1	1	0	1	Kanamycin
Gynecomastia	1	0	1	1	Ethionamide
Headache	1	0	1	1	Kanamycin
**Total**	**108**	**50(46.3)**	**58(53.7)**	**88(81.5)**	

### Statistical analysis

Data were entered into EpiData®, version 6.1 and analyzed in Stata®, version 11. Frequencies were used to compare differences in demographic and baseline clinical data between patients experiencing SADRs and those not as well as for describing the frequencies and range of SADRs. Significance of differences were tested by chi square or Fischer's exact.

## Results

**Figure 1:**
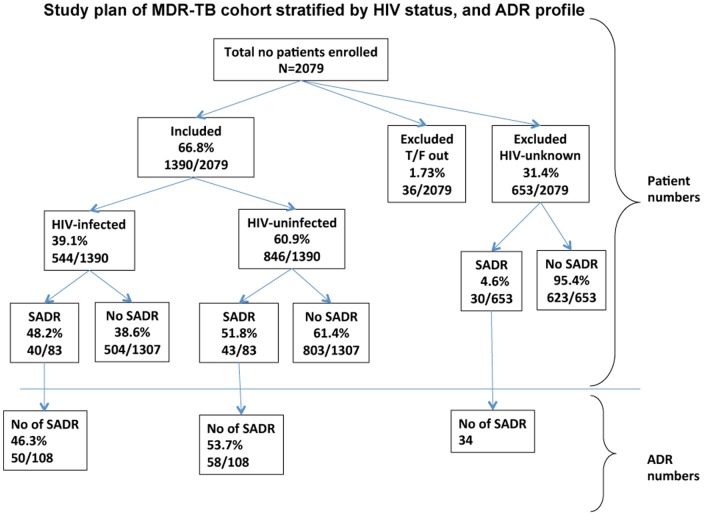
Study plan stratified by HIV status.

A total of 2079 patients were enrolled in the DOTS-Plus cohort. Of these, 1390 (66.8%) had a known HIV test result (Figure 1) and did not transfer between MDR-TB centres after cohort entry. Eighty three (6.0%) of patients experienced at least one SADR. Patients who did not experience SADRs (i.e. none, mild or moderate ADR's) were younger, had lower pre-treatment weights and consisted of more males, but these differences were not significant (Table 1). Numbers of patients experiencing SADRs was almost the same in HIV-uninfected patients 51.8% (43/83) and those infected 48.2% (40/83) (Figure 1). The number of SADRs experienced by patients was higher in HIV-uninfected patients 53.7% (58/108) compared to 46.3% (50/108) in the HIV-infected sub-group. The majority of the cases in our cohort had received previous TB treatment, presented with sputum smear-positive disease and had known radiographic findings (Table 2). There were higher rates (91.4% vs. 84.0%) of bilateral disease recorded for patients with SADRs compared to those with no reported SADRs (p = 0.189). More patients without SADRs were successfully treated 54.3% (662/1307) vs. 57.8% (48/83) although this was not statistically significant (p = 0.215). Likewise rate of default was lower in the cohort of patients experiencing SADRs 11/83 (13.3%) vs. 278/1307 (21.3%) p = 0.108.Overall there was no differences in treatment outcomes (p = 0.406).

**Table 2:** Baseline Demographic and Clinical Characteristics of patients with and without Serious Adverse Drug Reactions (SADR), N = 1390.

	No Serious ADR n = 1307 Mean (±SD)		Serious ADR n = 83 Mean (±SD)		P value (CI 95%)
Age (years)	n = 1292* 36.3 (10.6)		n = 83 37.3 (11.3)		0.425
Pre-Treatment Weight (kg)	n = 1171* 50.9 (10.5)		n = 72* 51.2 (11.3)		0.747
	Number	(%)	Number	(%)	
Male	n = 1304* 797	(61.1)	44	(53.0)	0.164
HIV-infected	504	(38.6)	40	(48.2)	0.083
MDR Classification					0.950
New MDR TB, Unknown Previous TB	35	(2.7)	2	(2.4)	
New MDR-TB, No Previous TB	88	(6.7)	6	(7.2)	
New MDR-TB, Previous TB	1184	(90.6)	75	(90.4)	
Radiographic Findings Total	935*		58*		
Unilateral Disease	150	(16.0)	5	(8.6)	
Bilateral Disease	785	(84.0)	53	(91.4)	0.189
Smear status Positive	n = 1164* 806	(69.2)	n = 74* 55	(74.3)	0.435
Culture status Positive (at treatment initiation)	n = 1226* 1051	(85.7)	n = 75* 64	(85.3)	0.866
Treatment Outcome					0.406
Cure	349	(26.7)	27	(32.5)	
Completed	313	(24.0)	21	(25.3)	
* Successfully treated*	*662*	*(54.3)*	*48*	*(57.8)*	
Failure	115	(8.8)	6	(7.2)	
Default	278	(21.3)	11	(13.3)	
Died	252	(19.3)	18	(21.7)	

While the majority of patients 63/83 (75.9%) experienced one SADR during their duration of treatment, 15/83 (18%) experienced two SADRs while five patients (6.0%) experienced a total of three. Decreased hearing, leading to withdrawal of the injectable was the most frequent reaction observed 42/108 (38.9%), followed by psychosis in 16/108 (14.8%), and the remainder of the 14 reactions being reported less than ten times (Table 1).

Cycloserine/terizidone, together with ofloxacin were the drugs implicated in those patients in whom psychotic side effects were experienced. Of the 16 SADRs which were psychotic in nature, 8 were observed during the intensive phase while the remainder were experienced during the continuation phase. The majority of all the other side effects occurred during the first four months of treatment (Table 1). Fifty side effects occurred amongst the 40 HIV-infected cases, and 58 amongst the HIV-uninfected cases. On average HIV- infected patients experienced 1.25 SADRs, with HIV-uninfected patients experiencing 1.35 although this was not statistically significant.

## Discussion

This is the first evaluation of SADRs among a cohort of HIV-infected MDR-TB patients, on standardised treatment regimens in South Africa in the absence of ARV therapy [12]. Six percent (83/1390) of cases in our cohort experienced a serious adverse drug reaction, and successful treatment outcomes (cure plus completion) were higher in those whom had experienced SADRs and fewer patients with SADRs interrupted their treatment (Table 1). The latter could be due to differences in management of patients with SADRs, which would have included increased emotional support which could otherwise have resulted in patients defaulting treatment or failing treatment.

We do not believe that being HIV-infected *per se* predisposes a patient to experience SADRs; and this study confirms that the rate and range of SADRs amongst HIV-infected ART naïve and uninfected patients are similar. However, HIV-uninfected cases experienced slightly more, (1.35) serious adverse drug reactions on average, compared to the 1.25 in HIV-infected cases.

There were relatively fewer SADRs experienced in our cohort compared to other MDR-TB treatment cohorts [7], [10], [14] despite our high HIV co-infection prevalence. These cohorts consisted almost exclusively of HIV-uninfected patients and had limited differentiation in the severity of drug reactions. Forty percent (42/108) of the SADRs experienced in our study were related to decreased hearing making it the most frequently reported SLD treatment related adverse drug reaction. This is in keeping with the findings of Torun, who recorded that 41.8% of patients who experienced ototoxicity necessitating the withdrawal of the culprit drug and Sagwa, (Namibia) whose study showed high numbers of hearing loss and tinnitus 70% (37/53) [14], [15]. This is in contrast to other investigators who found nausea and vomiting to be the most frequent SADR [6]–[8].

We identified SADRs through passive screening, which could be prone to clinical subjectivity or patient self-reporting/tolerance levels. The management of adverse drug reactions also depended on the clinician's access to other screening tests, e.g. audiology, ancillary treatment and other patient psychological support systems. The second most common adverse drug reactions were the psychotic episodes 16/108 (14.8%), and in contrast to the other drug reactions, occurred as often after the first four months of treatment as it did during the last 16 months.

We note several limitations in our findings. We have reported a relatively small number of ADR's compared to other studies but this could be due to the fact that we were only reporting on SADRs. We also may have missed a range of drug reactions that showed association with baseline clinical factors or with specific treatment outcomes. Furthermore, it could not be guaranteed that clinicians from all the MDR-TB centres were consistent in their reporting of SADRs despite close monitoring, ongoing education and encouragement to report ADRs. Furthermore, excluding a number of MDR-TB patients without any HIV information may have biased our results. Access to CD4 counts of the HIV-infected cases could have improved the description of the cohort and allowed us to explore those associations. During the DOTS-Plus study period the case loads in the MDR-TB treatment centres were much lower than they have been in the past few years [10], [13] and therefore most cases were able to be hospitalised for a minimum of four months or until culture conversion had taken place. During the patients' out-patient phase of treatment there is a high possibility that the occurrence of SADRs were underreported. Our findings should not be generalised to settings with other management approaches to MDR-TB management, e.g. outpatient or community-based management.

Towards the end of the DOTS-Plus study and onwards, HIV-infected MDR-TB cases had access to anti-retrovirals with overlapping toxicities of the two regimens being recognized as a challenge in universal access of combined therapy [12]. For cases on co-therapy, our findings provide baseline data on the range and frequency of SADRs due to second-line MDR-TB drugs, when they can be expected as well as providing clinicians with a guide for designing and implementing appropriate interventions.

The observation that the majority of the SADRs occurred during the first four months of treatment has ramifications for MDR-TB management in South Africa as the country moves towards decentralised care with treatment of patients nearer to their homes and where they can receive support from the community in which they reside. Similarly, our findings showing that an HIV-uninfected case could experience more adverse drug reactions than an HIV-infected case, means that HIV-uninfected cases should not be neglected from screening.

These models of care should include tools specific for the vigilant screening of serious adverse drug reactions by all clinicians as well as an appropriate and timely response once identified. An integral part of the successful management of adverse drug side effects within these models, especially for those co-infected, will be the improved treatment literacy of patients, and the equipping of close contacts and treatment supporters on providing the necessary treatment support to ensure case holding and in turn improved treatment outcomes.
